# Influenza-Omics and the Host Response: Recent Advances and Future Prospects

**DOI:** 10.3390/pathogens6020025

**Published:** 2017-06-10

**Authors:** Joshua D. Powell, Katrina M. Waters

**Affiliations:** 1 Biomarkers Division, Battelle Memoria Institute, Aberdeen, MD 21001, USA; 2 Earth and Biological Sciences Directorate, Pacific Northwest National Laboratory, Richland, WA 99352, USA; Katrina.Waters@pnnl.gov

**Keywords:** influenza, transcriptomics, proteomics

## Abstract

Influenza A viruses (IAV) continually evolve and have the capacity to cause global pandemics. Because IAV represents an ongoing threat, identifying novel therapies and host innate immune factors that contribute to IAV pathogenesis is of considerable interest. This review summarizes the relevant literature as it relates to global host responses to influenza infection at both the proteome and transcriptome level. The various-omics infection systems that include but are not limited to ferrets, mice, pigs, and even the controlled infection of humans are reviewed. Discussion focuses on recent advances, remaining challenges, and knowledge gaps as it relates to influenza-omics infection outcomes.

## 1. Introduction

“Omics” data refers the large-scale acquisition of specific “features” in a biological sample, including genes (genomics), gene expression in the form of mRNA (transcriptomics), or protein expression (proteomics) that may include specific post-translational modification events (phosphoproteins, glycosylation, etc.). Applied to studies of pathogens, -omics analyses can identify hundreds to thousands of signature features that characterize temporal microbial and host response events during the course of infection. Features of interest are dependent on the experimental system and hypothesis, but -omics studies often interrogate the expression level of RNA or protein for a target of interest in the infected sample versus an uninfected reference control. After data is acquired, bioinformaticists must make sense of these microbial and/or host changes to identify infection-related pathways. Additionally, promising novel host gene antiviral candidates that are impacted by the infection process may also be of interest. Finally, it may be of interest to elucidate how genetic variants in either the host or the pathogen itself may impact immune responses. As next-generation sequencing (NGS) technologies such as shotgun transcriptomic RNA sequencing (RNA-seq) replace more conventional microarray analysis, and proteomic approach platforms become more sensitive, quantitative assessment should also become more reliable. In summary, -omics-driven experiments represent a very powerful tool to study the host response to infection, but data interpretation is critical to ensure that meaningful conclusions are reached.

This review focuses on the numerous influenza A virus (IAV) omics-related studies of host responses that have been published over the last two decades. While vaccination strategies exist to combat the seasonal spread of IAV, these viruses can mutate and undergo genetic reassortment that can evade vaccines and other therapeutics. Of particular concern is that not just humans, but wild and domesticated birds can spread IAV, including recent highly pathogenic bird flu isolates of H7 and H5 designation that cause high mortality rates in infected humans. Additionally, IAV is also endemic in pigs, with the 2009 H1N1 global pandemic caused from a swine-to-human spillover event. Monitoring multiple reservoirs of IAV for zoonotic spillover events into humans therefore requires the characterization of IAV isolates on a continual basis and to define host responses—including -omics approaches—in multiple species. As IAV strains evolve and sequence information from the IAV cannot currently determine pathogenicity, -omics platforms can be a powerful tool to compare emergent IAV strain data with previously circulating strains for the purpose of threat characterization.

**Figure 1:**
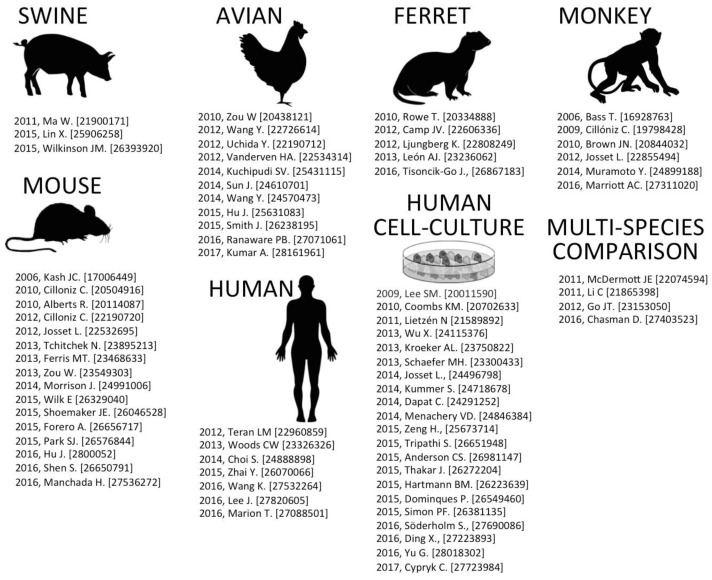
Influenza-omics publications categorized by infection type. Noted is the year of publication, the first author, and the PubMed database identifier number (PMID).

In the last ten years, mass spectrometry instrumentation and methodology advancements have led to considerable knowledge of the host proteomic response to viral infection (for a recent review, see [1]). The increasing popularity of NGS technologies such as RNA-seq, in combination with further advances in microarray profiling technology has also increased our transcriptomic-RNA understanding of virus–host interactions. As -omics capabilities become cheaper and the software to interpret data becomes more user-friendly, the number of laboratories that can perform these data-intensive studies will likely increase. The purpose of this review is to assess both proteomic and transcriptomic findings in the context of IAV infection. In the following sections, recent advances and future prospects are discussed. Figure 1 summarizes notable published -omics experiments, many of which are discussed in the proceeding sections.

## 2. Host Response Models of IAV Infection

A number of published data sets examining host-IAV interactions and subsequent meta-analysis are summarized in Figure 1. Numerous -omics influenza studies have been funded by the National Institute of Allergy and Infectious Diseases (NIAID) Systems Biology for Infectious Diseases Research program [2]. NIAID-related data sets from these virus studies are publically accessible through the Influenza Research Database (www.fludb.org) [3]. Additionally, the National Center for Bioinformatics (NCBI) Gene Expression Omnibus (GEO) serves as a public repository for various high-throughput -omics experimental data [4]. These data include microarray-based experiments measuring mRNA, genomic DNA, and protein abundance. GEO also provided a repository for non-array techniques such as serial analysis of gene expression (SAGE) and mass spectrometry proteomic data. Collectively, GEO allows researchers from all over the world access to data for analysis on various software programming packages [5].

### 2.1. Swine-IAV Infection

Swine influenza viruses (SIV) have the zoonotic potential to cause global pandemics in humans, such as the 2009 H1N1 pandemic (pH1N1) that spread across the globe [6]. Swine are considered a mixing vessel for novel influenza reassortment, as pigs have the capacity to support influenza strains of human, swine, and avian origin. While swine influenza is a public health concern, compared to humans, we have a limited understanding as to swine host responses to influenza infection at the -omics level [7,8].

In 2011, Ma and co-workers [9] performed a whole-genome analysis using swine-specific microarrays to compare transcriptomic pig lung responses of human origin pH1N1 to that of either swine origin pH1N1 and a classical SIV strain (IA30). pH1N1 human and pig viruses caused clinical symptoms and upregulation of genes related to immune and inflammatory responses at day 3 post-infection (pi) that were not evident in the classical SIV strain. At day 5 pi, expression of cell death and lipid metabolism genes substantially differed in pH1N1-infected pigs versus those of IA30 infection. Building on their earlier work, in 2012 Michael Katze’s lab also compared their human swine microarray pH1N1 (CA 04/09 strain) data to mice and macaque lung responses after infection with the same virus [10]. The authors noted similar clinical courses of infection progression, but they also found significant differences between host species in lipid metabolism and inflammatory gene responses. In particular, CA 04/09 virus significantly altered the expression of cholesterol homeostasis genes (LXR/RXR) in swine and mice, and vitamin D receptor genes in macaques. In total, 53 genes were found to be differentially regulated between species.

Notable swine -omics studies include Lin and co-workers’ [11] assessment of swine H1N1 IAV and *Streptococcus suis* serotype 2 (SS2) co-infection through microarray analysis. These authors found that co-infected pigs had altered interferon and interleukin receptor expression, as well as differential expression for several chemokine and chemokine receptor genes, along with the differential expression of five tumor necrosis factor (TNF) receptor superfamily genes versus either IAV or SS2 infection alone. Li and co-workers [12] using A/swine/Hubei/101/2009 (H1N1) also studied influenza infection in swine lungs at day 3 and day 7 pi through microarray. These studies confirmed that toll-like receptor genes (TLRs) and interferon-induced genes (ISGs)—which are involved in anti-viral signaling—were heavily impacted after infection. Additionally, Wilkinson and colleagues [13] also used microarray analysis to compare infection outcomes in low vs. high birth weight litters with swine H3N2 (Tx98) that resulted in 48 differentially expressed genes including IL6, IL8, and CCL2. These authors also characterized transcriptomic responses based on disease severity in pigs. Lastly, while there are minimal large-scale in vivo proteomic experiments, Zhu and co-workers [14] have experimentally infected porcine macrophages with two different swine H1N1 strains. Using 2D-gel electrophoresis and MALDI-TOF MS/MS, the authors identified 13 up-regulated and 21 down-regulated protein spots associated with molecular biosynthesis and heat shock proteins. Considering the global concern of IAV circulation in pigs, additional -omics data sets should prove useful to help elucidate potential host factors that are impacted by infection and to identify those IAV circulating strains that could pose a threat to human health.

### 2.2. Avian-IAV Infection

Numerous avian IAV subtypes circulate in domestic and wild birds, with occasional spillover into humans. Of particular concern are H5N1 and H7N9 that have been transmitted from poultry to humans in Asia, resulting in infections with high mortality rates [15]. Using a chicken 44 K Agilent microarray, Wang and colleagues studied H5N3 lung infection in broiler chickens and found 508 differentially expressed mRNAs compared to mock-infected chickens [16]. Building on these earlier studies, the same group in 2014 used a next-generation sequencing RNA-seq platform approach with the same H5N3 virus to look at infection in two inbred chicken lines—one resistant to and one susceptible to IAV infection. The authors noted that their bioinformatics data analysis inferred that the hemoglobin gene family and cell adhesion molecule signaling pathways play prominent roles in IAV disease resistance in chickens. A recent microarray study comparing highly-pathogenic H5N1 versus low-pathogenic H9N2 in the chicken lung provided valuable insight into inflammatory/cytokine host gene response differences related to infection outcomes [17]. In particular, the authors found 5550 differentially expressed host genes after H5N1 infection and 2992 host genes after H7N9 infection at 6 days pi, respectively. Of note, a 2012 study took a similar approach using a Nimblegen chicken genome array to analyze 38,681 host genes in response to low and high pathogenic recombinant IAV infection in chickens [18]. The authors wanted to assess genes related to survivability, and deduced that CD274, RNF19B, OASL, ZC3HAV1, PLA2G6, GCH1, and USP18 were important factors for regulating IAV severity. Finally, Smith and co-workers have explored the transcriptomic sequencing differences between ducks and chickens in high (H5N1) and low (H5N2) pathogenic IAV [19]. The authors note that unlike ducks, chickens lack the retinoic acid-inducible gene I (RIG-I) virus sensor gene, which partially explains severity differences after IAV infection between species. Additionally, the authors note that differences in the expression of the interferon-induced transmembrane protein (IFITM) family may restrict infection in ducks to a greater extent than in chickens.

Similar to swine studies, there is also limited proteomic analysis data available for avian species, but Sun and co-workers have identified 38 proteins using 2D-DIGE (differential gel electrophoresis) followed by MALDI-TOF/TOF-MS in the trachea of IAV-infected chickens [20]. The authors found that two annexin proteins (ANXA1, ANXA2), and a heat shock protein (HSPB1) were differentially expressed in infected chickens. Of note, Zhou and co-workers [21], using a similar approach identified 18 up-regulated proteins and 13 down-regulated proteins in the brains of H5N1 infected chickens, as this highly pathogenic virus is known to cause neurovirulence. The most notable protein changes that authors found occurred within cytoskeleton and ubiquitin-proteasome pathway related proteins.

### 2.3. Ferret-IAV Infection

Ferrets are considered to be an ideal small animal model for IAV infection, as they have similar sialic acid characteristics for IAV binding to that of humans [22]. Additionally, ferrets are ideal for IAV in vivo studies, as they exhibit clinical symptoms of infection and can transmit IAV through sneezing [23]. While the ferret model is used for pathogenesis, transmission, and virulence studies, the host response at the molecular level has only resulted in a handful of publications. Last year, Tisoncik-Go and co-workers [24] used an integrated high-throughput -omics approach to study the difference between the pH1N1 CA04/09 strain and the highly virulent 1918-H1N1 virus in infected ferrets. In particular, the authors focused on the lipids, metabolites, and proteins in respiratory compartments for highly virulent versus less virulent outcomes in ferret lungs. These findings identified 488 unique lipids in the lung and 191 lipids in the trachea, along with LC/MS analysis identifying 4811 proteins in the lung and 4060 proteins in the trachea of infected ferrets. Key infection findings included significant abundance changes in phospholipids (diacylglycerophosphocholine and diacylglycerophosphoethanolamine species) known to be major constituents of pulmonary surfactant and phospholipid precursors that can be cleaved to form arachidonic acid that would impact IAV pathogenesis. Additional studies have also characterized the influenza “infectome” through NGS and microarray analysis of ferret lung and lymphoid tissue with H1N1 (A/Mexico/4108/2009) [25]. This study identified 2926 genes that were significantly upregulated, and 637 genes that were downregulated. Interferon-stimulated genes were markedly upregulated, including antiviral responses genes CXCL10, OAS1, IRF1, and RSAD2, as well as various cytokine and chemokine genes related to immune cell recruitment. Ferret-specific microarrays were also used in a 2012 study to assess distinct infection profiles for three differing H1N1 influenza strains from the blood of ferrets [26]. In particular, authors identified a gene expression profile consisting of 31 probes that could classify samples based on both strain and severity of disease. Finally, in one of the earlier ferret -omics studies by Camp et al. [27], the authors identified more than 19,000 partial ferret transcripts, including more than 1000 gene orthologs known to be involved in the innate and the adaptive immune response through de-novo transcriptome sequencing.

### 2.4. Monkey (Macaque)-IAV Infection

The macaque model (rhesus and cynomolgus) for IAV infection has resulted in the generation of insightful -omics data sets. A 2015 review summarized the historical significance of using the nonhuman primate model for IAV infection [28]. One of the earliest macaque -omics studies in 2006 compared uninfected versus infected monkeys using H1N1 A/Texas/36/91 (Tx91) [29]. In this study, microarray profiling for approximately 18,000 genes was assessed, and virus-related interferon and cytokine responses were found to be sustained throughout the course of the infection in both lung and blood samples. In this same study, proteomic LC-MS-MS was also assessed from monkey lungs and blood, resulting in a total of 14,100 peptides and 3548 proteins that were collectively identified. Proteins related to infection included several well-known interferon-induced proteins relevant to neutrophil and monocyte/macrophage function. In 2010, a proteomics study compared highly pathogenic H5N1 and 19-H1N1 versus the less virulent Tx91 seasonal influenza strain [30]. Over 35,000 peptides representing 4219 proteins were identified, with 400 increased proteins and 258 decreased proteins associated with viral infection. Classical protein responders of viral infection included MX1, MDA5, OAS2, ISG15, and RIG-I. In 2009, Cillóniz and co-workers [31] used whole-genome microarray analysis to compare highly pathogenic avian H5N1 to the pathogenic 1918-H1N1 virus. Higher tissue pathology was evident in 1918-H1N1 infected macaques, which demonstrated upregulation of key components of the inflammasome, including NLRP3 and IL-1beta. Interestingly, these genes were downregulated in animals infected with the H5N1 virus, which resulted in a less severe infection compared to the 1918-H1N1 virus. A 2012 study compared pH1N1 CA04/09 infection in aged versus young macaques [32]. Aged animals had higher viral titers and increased cytokine expression in bronchoalveolar lavage (BAL) fluid through use of a multiplex ELISA. Follow up whole-genome microarray experiments—which found 709 genes differentially expressed between young adult and aged macaques—confirmed increased inflammatory gene expression in aged animals as well as the enrichment of protective genes associated with naïve T or B cells in younger animals. McDermott and co-workers have comparatively assessed H5N1 transcriptomic infection outcomes in Calu-3 human cells, mouse lung, and the macaque lung (discussed further in Section 2.5) [33]. Additionally (and as mentioned earlier), swine, mouse, and macaque lung infections with pH1N1 have also been comparatively assessed at the transcriptome level [10]. Finally, a proteomic approach was used for pH1N1-infected cynomolgus macaques that identified over 100 differentially expressed proteins in the BAL versus uninfected controls [34]. These authors found that there was a significant increase at day 7 relative to day 5 pi in abundance of interleukins (ILs) including IL1B, IL2, IL4, IL5, IL6, IL10, and IL13, as well as proteins associated with the interferon response versus uninfected monkeys.

### 2.5. Mouse-IAV Infection

Extensive -omics analysis has been investigated using the mouse model for influenza infection. One of the earliest studies in 2006 compared host responses of the 1918-H1N1 virus versus the Tx91 season strain virus, in addition to Tx91 reverse-engineered to contain either the 1918-H1N1 HA and NA genes (2:6 1918), or the 1918 HA, NA, M, NP, and NS genes (5:3 1918). In 1918-H1N1, there was increased macrophage and neutrophil recruitment after infection compared to the other viruses. Additionally, increased and earlier expression of cytokine and chemokine-associated genes in mouse lungs infected with 1918-H1N1 was evident through transcriptomic-microarray analysis. In 2015 the same lab performed additional transcriptomic profiling and narrowed down the pathogenicity determinant to the PB2 segment for 1918-1H1N1 [35]. Extensive transcriptomics analysis has also been performed comparing pathogenicity differences between H5N1 and H1N1-1918 in mouse lungs [36]. The authors found that unlike H1N1-1918, H5N1 disseminated to extrapulmonary organs with upregulation of inflammasome genes more so than H1N1-1918. In addition, the authors note that H5N1 also upregulated the expression of tumor necrosis factor alpha (TNF-α)—a potent inflammatory molecule; IFN-γ; eukaryotic initiation factor 2 AK2 (eIF2AK2); protein kinase RNA activated (PKR); and additional chemokines and inflammation-related genes. Other notable mouse transcriptomics studies include those of Zou and co-workers [37], who used an NGS RNA-seq approach to assess pH1N1 versus swine origin IAV outcomes in mouse lungs. Interestingly, the authors found that the swine-origin virus caused higher mouse morbidity with numerous cytokines and interferon-stimulated genes having higher expression level in swine H1N1 infected groups. An additional RNA-seq study assessed highly pathogenic H5N8 versus H5N1 in mouse lungs, and the authors found higher pathogen-related transcripts for H5N1-infected mice at day 3 and 7 [38]. The authors noted that inflammation pathways were elevated in H5N1-infected mice versus H5N8, which was consistent with higher observed viral titres and virulence. Finally, Morrison and co-workers [39] used microarray analysis and studied outcomes in the BALB/c mouse strain with pH1N1 and three avian strains (H5N1, H7N9, and H7N7). Avian viruses were more pathogenic than pH1N1, with host gene signatures reminiscent of 1918-H1N1. Coagulation genes and LXR/RXR pathway genes were highly upregulated by pH1N1, but not by the avian viruses; the authors state that this finding suggests that the avian viruses were potentially suppressing gene expression in these two pathways. Of note, the authors also assessed the host responses that could be targeted using FDA-approved drugs that could potentially be repurposed to treat H7N9 infection using six potential therapeutics.

While mouse models of IAV infection can be insightful, not all mouse strains are equally susceptible to infection. In 2010, Alberts and co-workers [40] infected a resistant strain (C57BL/6J) and a highly susceptible mouse strain (DBA/2J) with H1N1-PR8 and performed genome-wide microarray analysis. The magnitude of immune response genes was significantly higher in DBA/2J compared to C57BL/6J, along with several immune response genes exclusively regulated in DBA/2J. The authors also noted a region of chromosome 3 that was significantly different in the expression for both strains. A similar study comparing two strains with disparate IAV susceptibility, but using NGS RNA-seq, found the Irf7 gene to be a virulence determinant [41]. Of interest, researchers at the University of North Carolina have generated a panel of genetically diverse mouse strains referred to as the Collaborative Cross (CC) (for review see [42]) and compared them to pre-cross strains for H1N1-PR8 infection outcomes [43]. By conducting quantitative trait loci (QTL) mapping in combination with transciptomic-microarray profiling, the authors identified a novel allele within the anti-influenza Mx1 gene that was a major determinant of influenza pathogenicity.

Proteomic approaches to assessing influenza infection in mice are limited compared to transcriptomics, however there is recent progress that may reflect increased capabilities in this field and indicates the potential for future work. Last year, Hu and co-workers [44] compared a high versus low pathogenic H5N1 strain in mouse lungs using isobaric tags for relative and absolute quantitation (iTRAQ) coupled LC-MS/MS method. The highly-pathogenic H5N1 strain up-regulated proteins corresponding to inflammatory response, cell death, reactive oxygen species production, and complement response more so that the low-pathogenic H5N1 strain. Another study from last year identified over 1600 proteins collectively from days 1, 7, and 10 pi with H3N2 A/Hong Kong/X31, and found decreased levels of several cell junction proteins but increased levels of tissue metalloproteinase MMP9 [45]. In a separate analysis, Zhao and co-workers [46] performed proteomics on 2D differential gel electrophoresis (DIGE) protein spots for H5N1-infected mouse lung tissues using a lethal versus a non-lethal strain. Of the 39 differentially regulated proteins for infected versus uninfected controls, 13 were different between highly pathogenic H5N1 versus low pathogenic H1N1. Of note, five virus-related proteins (TGTP, IFIT3, IFIT3-L, LCP1–1, and LCP1–2) were upregulated as soon as day 1 pi in the lethal virus-inoculated group, but did not show appreciable upregulation until day 3 pi in the non-lethal group. Their results suggest that proteomic approaches can distinguish the lethality of the influenza strain in a mouse model within 24 h of infection.

### 2.6. Human-IAV Infection

Human influenza trials and the accompanying -omics data sets have been documented, but in a rather limited manner. With human trials, there are obvious ethical restrictions to using high pathogenicity influenza strains to infect volunteers, and therefore only safer seasonal IAV strains are permitted. Furthermore, significant harm to the trial participant has to be avoided, therefore preventing invasive biopsies with usually only serum blood draws and nasal secretion collection permitted. Nevertheless, these studies often enable valuable protracted longitudinal sampling on the same patient. Building upon previous microarray data sets [47,48], Wang and co-workers [49] applied a multivariate estimation technique (MSET) for H3N2 and H1N1 human infection. The authors were able to monitor the acute phase of infection and quantify the predicted health outcome of infection from the blood of patients. This technique could prove useful, as it could potentially diagnose influenza severity before the patient shows signs of sickness.

Besides clinical trials, surveillance of active infection has proved invaluable for acquiring -omics data. For example, in 2009–2010, researchers enrolled 1610 healthy adults in a study where the volunteers self-reported and blood draws were performed within 48 h of fever onset. This was followed up with additional sample acquisition at day 2, 4, 6, and 21. In total, 64 influenza A, 9 influenza B, 32 rhinovirus, and 24 patients with fever but no viral agent were analyzed. Collectively, these data sets provide a wealth of information to understand the systemic response to naturally acquired respiratory infection through the analysis of acute versus recovery gene expression [50]. An additional study included a novel proteomics technology referred to as SOMAscan to quantify 1030 different human proteins from the human mucosa (nasal lavages) of influenza patients for the purpose of using this technology as a biomarker tool [51]. The authors noted that of the 160 differentially expressed proteins between infected versus uninfected patients, many were associated with the recruitment and activation of memory T cells, most notably CXCL11, CXCL10, and IL-16.

### 2.7. Human Cell Culture-IAV Infection

Human tissue culture cell lines provide a convenient *in vitro* means to study host responses to influenza infection. While cell lines cannot recapitulate some clinical aspects of natural infection, they can still be informative. As discussed earlier, comparisons between human cells, mice, and non-human primates do show conserved innate host responses to highly-pathogenic H5N1 [33]. Due to convenience and cost, numerous studies at the -omics level have therefore implemented a cell line approach. Another benefit of cell line use is the potential for consistency between experiments and even labs when obtaining -omics data. For example, certain human lung epithelial cell lines such as Calu-3 and A549 that are widely available allow researchers to compare their new findings to prior data sets, as an immortalized cell line is derived from a single donor. The benefits of cell lines, as well as some of the drawbacks of their use are saved for the discussion section.

In 2014, the Katze lab at the University of Washington studied the transcriptomic responses of human Calu-3 cells infected with either avian-origin H7N9, H5N1, H7N7, or human seasonal H3N2 [52]. The H7N9 Ahuni/01/13 strain was found to more closely resemble the seasonal H3N2 in transcriptomic responses compared to the other two avian strains. This finding suggests that the H7N9 has at least been partially adapted to humans. Importantly, using genome-based drug repurposing approaches, with the Ingenuity Knowledge Database and the Connectivity Map (Cmap) data driven tool, the authors found that already-FDA-approved kinase-inhibitors could potentially be repurposed as novel drug targets based on -omics data. This analysis also suggested that SB-203580 and genistein may revert the host response to all four IAVs tested. These studies infer the potential for critical pathways for IAV adaptation that could be targeted using therapeutics. Of note, microarray findings from this study were in agreement with a separate Calu-3 cell infection study that found similar transciptomic profiles for H7N9 and a human H3N2 virus compared to two other avian strains, suggesting human adaptation for H7N9 [53]. These authors also noted more efficient replication and viral titers for this partially adapted H7N9 bird flu strain at the human temperature of infection (37 °C) versus the avian favorable infecting 33 °C temperature.

Proteomic approaches have also been used on immortalized lung cell lines. Coombs and co-workers [54] have used a stable isotope labeling amino acid (SILAC) approach in cell culture and high throughput 2-D HPLC mass spectrometry to look at uninfected and infected A549 cells with the widely used A/Puerto Rico/8/34 H1N1 strain. Of the 4689 proteins identified, 280 were either significantly up- or down-regulated. Gene ontology pathway analysis indicated numerous functional profiles that were impacted, including host cell immunity, cell adhesion, metabolism, and signal transduction. The authors noted that key proteins upregulated after infection included Mx1, LTF, and VIM, while those that were downregulated included ERC1, L1CAM, and CTNNB1.

Infiltrating cells in the lung (e.g., macrophages and monocytes) can also have unique signatures after IAV infection. In a 2009 -omics study by Lee and co-workers [55], macrophages infected with high pathogenic H5N1 versus less pathogenic H1N1 led to stronger type I interferon and TNF-alpha expression. A recent proteomics study also with infected macrophages revealed that IAV can cause robust secretions of proteins, including those associated with antiviral cytokines, copper metabolism Murr-1 domain proteins, and autophagy-related proteins [56]. Lastly, in 2011, a proteome and secretome study using cell fractionation and a 4-plex iTRAQ approach showed dramatic changes in mitochondrial and nuclear proteomes in response to Udorn/72 and Beijing/353/89 H3N2 infection [57]. In particular, the authors found cytoplasmic leakage of lysosomal proteins, including cathepsins, results in inflammasome activation, and apoptosis of the macrophage cell. In summary, macrophage studies are informative, as similar to the lung epithelium, macrophages can provide a unique biomarker platform for measuring influenza infection severity. In the discussion section, additional cell types and ways to provide more relevant cell culture platforms to study influenza -omics are discussed.

## 3. Discussion

In order for -omics data to be meaningful, the study authors must relate their data sets to that of the underlying biology. Various software packages that run within R or Python programming languages are currently used for analysis. The stand-alone Ingenuity Pathway Analysis (IPA) platform available through QIAGEN is the most popular -omics software approach for elucidating the upstream biological causes and probable downstream effects for gene targets of interest [58]. IPA software has been cited in thousands of articles for the analysis, integration, and interpretation of data derived from proteomic and transcriptomic experiments. While software packages such as IPA can help a researcher interpret their own data, there is still a level of ambiguity in comparing influenza -omics data from two or more different studies. The particular IAV strain, time points chosen, and the starting infection dose—referred to as multiplicity of infection (MOI)—are all major considerations when comparing results from one study to those of another. For *in vivo* studies, additional considerations such as the age of the animal, particular animal strain, volume of virus inoculum, and cell/tissue collection method also impact the -omics findings. Unfortunately, while determining what signifies a statistically upregulated or downregulated RNA or protein is straightforward, study design differences that reflect an -omics “snapshot” of slightly differing phenotypes and methodologies may lead to differing conclusions.

As reviewed in [59,60,61], lethal IAV infection leads to cytokine dysregulation and upregulation of inflammatory host genes during virus spread. However, comparing severe versus mild infection does not lead to wide-scale differences in cytokine/chemokine gene induction, but rather the magnitude of the response. For example, when mice were infected with H5N1 IAVs of different virulence, the host responses differed primarily in magnitude and velocity, rather than if a host gene was activated or not [62]. While there is no magic bullet gene at one time point to tell you how virulent an IAV strain may be, a defined series of influenza-specific signature genes can provide a comprehensive view into IAV pathogenicity. For instance, a meta-analysis from 12 studies comparing IAV strains as well as SARS coronavirus allowed Chang and co-workers [63] to show a relationship between the expression of 57 chemokine subnetwork genes and influenza severity in mice. This type of meta-analysis is promising so future researchers can hopefully analyze a handful of genes to deduce the pathogenicity of an emerging IAV strain.

As prior virus systems biology reviews have noted [64,65,66], -omics approaches often seek to define virus–host immune responses to better design therapeutics such as antivirals and better vaccine strategies. The field of virus -omics is an evolving discipline that has increased in complexity and popularity over the last decade. Comprehensive proteomics and transcriptomics influenza studies not only require laboratory competency under biosafety level-3 (BSL-3) conditions, but competency in computational modeling to handle the sheer amount of data generated. Collaboration between research groups is paramount to ensure adequate resource sharing and data availability to others in the virology community. Lastly, while the field of virus -omics can be costly, the high-throughput molecular techniques and computational tools used in these studies should continue to advance so as to allow more research to contribute to the field of systems virology.
